# Next Generation Sequencing Identify Rare Copy Number Variants in Non-syndromic Patent Ductus Arteriosus

**DOI:** 10.3389/fgene.2020.600787

**Published:** 2020-11-12

**Authors:** Bo Chen, Aiping Hou, Lin Zhao, Ying Liu, Xin Shi, Bowen Du, Yu Yu, Pengjun Zhao, Ying Gao

**Affiliations:** ^1^Department of Pediatric Cardiology, Xin Hua Hospital, School of Medicine, Shanghai Jiao Tong University, Shanghai, China; ^2^Department of Pediatric, Shidong Hospital, Shanghai, China

**Keywords:** congenital heart defects, patent ductus arteriosus, whole-exome sequencing, copy number variants, pathogenesis

## Abstract

Patent ductus arteriosus (PDA) is a common congenital cardiovascular malformation with both inherited and acquired causes. Several genes have been reported to be related to PDA, but the molecular pathogenesis is still unclear. Here, we screened a population matched cohort of 39 patients with PDA and 100 healthy children using whole exome sequencing (WES). And identified 10 copy number variants (CNVs) and 20 candidate genes using Gene ontology (GO) functional enrichment analysis. In gene network analysis, we screened 7 pathogenic CNVs of 10 candidate genes (MAP3K1, MYC, VAV2, WDR5, RXRA, APLNR, TJP1, ERCC2, FOSB, CHRNA4). Further analysis of transcriptome array showed that 7 candidate genes (MAP3K1, MYC, VAV2, APLNR, TJP1, FOSB, CHRNA4) were indeed significantly expressed in human embryonic heart. Moreover, CHRNA4 was observed the most important genes. Our data provided rare CNVs as potential genetic cause of PDA in humans and also advance understanding of the genetic components of PDA.

## Introduction

Non-syndromic patent ductus arteriosus (PDA) is a common congenital heart defect (CHD) (Hoffman and Kaplan, 2002). The incidence of PDA in different studies varies from about 2/10000 to 8/1000 live births in term infants, accounting for about 10∼21% of CHD (Hoffman and Kaplan, 2002). Persistent patency of the ductus arteriosus(DA) lead to pulmonary arterial hypertension, pulmonary edema, infective endocarditis, and congestive heart failure in humans (Garcia, 1975). The DA is an arterial vessel that shunts blood flow away from the lungs during fetal life (Vettukattil, 2016). It shut down functionally in 12 to 18 h after birth and anatomically in 2 to 3 weeks and then established a mature circulation pattern, which is the result of a long-term and dramatic vascular remodeling process (Clyman et al., 2012). If DA has not been closed in premature infants for more than 1 year, it is termed persistently patent ductus arteriosus (PDA) (Clyman et al., 2012). This process is complex including migration of neural-crest-derived cells into the subendothelial space, transformation to vascular smooth muscle cells (VSMCs), extracellular-matrix accumulation and formation of subintimal cushions. The final steps include contraction of smooth muscle with an increase in vasoactive peptides and a decrease in prostaglandin E2 levels.

So far, only a few genes have been proved to be the pathogenic genes of PDA. Satoda et al. (2000) identified the first genetic cause of PDA called transcription factor AP-2 β (TFAP2B) in patients with Char syndrome. The syndrome is characterized by PDA, facial dysmorphology, and fifth-finger clinodactyly (Satoda et al., 2000). Dagle et al. (2009) used genetic linkage analysis found two important pathogenic genes associated with the presence of a PDA: TFAP2B (rs987237: G allele) and TNF receptor-associated factor 1(TRAF1) (rs1056567: T allele). Patel et al. (2016) used transmission disequilibrium test found a DNA variation in TGF-β Receptor II (TGFBR2) associated with PDA called rs934328. However, the molecular genetic mechanisms are still largely unknown.

Copy number variants (CNVs) is defined as an increase or decrease of the copy number of large segments of the genome with a length of more than 1 kb, which is mainly manifested by the deletion or duplication at the submicroscopic level (Pinto et al., 2011). In recent years, large-scale CNVs have seen increased attention and play a key role in the pathogenicity of CHD (Sailani et al., 2013). CNV identification could be used as a new method for the new pathogenesis genes of PDA. It is not reported previously.

In our study, we identified 10 rare CNVs in 39 patients with PDA using whole exome sequencing (WES). We further identified seven potential candidate genes involved in several pathways reported to be related to heart development: Notch signaling pathway, or vascular smooth muscle development. The aim of our study was to determine the role of rare CNVs in PDA and explore distinguish the genetic mechanism.

## Materials and Methods

### Study Population

The study cohort enrolled unrelated 39 patients with PDA from the Xinhua Hospital Affiliated to Shanghai Jiao Tong University School of Medicine with ages ranging from 2 months to 13 years. Diagnoses were confirmed by echocardiography, cardiac catheterization, computed tomography, and other medical recordings. And 39 children included in this study were full-term infants. The control group consisted of 100 healthy children, aged 2 months to 13 years, who were excluded from cardiac malformation by echocardiography. The patients showing multiple major developmental anomalies, developmental syndromes, or major cytogenetic abnormalities were excluded. The study protocol was approved, and the ethical approval was given by the medical ethics committee of Xinhua Hospital.

### Whole Exome Sequencing and CNV Determination

The genomic DNA of participants was extracted from blood samples by using the QIAamp DNA Blood Mini Kit (QIAGEN, Germany) and then stored at −80°C. For all samples, we performed whole exome sequencing (WES) to detecting copy number variations (CNVs). Then the data was filter by HiseqTM Sequencer to get clean data (removing the adaptor sequences, reads with >5% ambiguous bases (noted as N), low-quality reads containing more than 20 percent of bases with qualities of <20 and sequences with reads less than 75 bp in length). Then the clean data was mapped to 1000 Genomes Project (Human genome Version human_glk_v37) utilizing BWA-mem. Duplicated reads were marked and removed by PICARD software.

Then we used CNVkit (Talevich et al., 2016) to calculate the CNV in the WES analysis. By comparing the results with the known CNVs in the Database of Genomic Variants (DGV^1^), common CNV was distinguished from rare CNV. Genomic variants were filtered on the basis of six factors: (1) CNV >500 kb, but <5 Mb in size; (2) average depth of sequencing >54; (3) Z-score of average depth ≥2.0; (4) present at <0.1% frequency or not found in the DGV; (5)The gene covered by CNV is protein coding; (6) no sample has detected CNV variation in control group, while the presence of at least 60% or more variation CNV samples (samples ≥24) in the case group (Hanemaaijer et al., 2012). Then, we used χ2 and Fisher’s test to compare the statistical differences between the patients and controls. *P*-value < 0.01 was statistically considered significant. Using R programming software (Chan, 2018) with version 3.5.0 to realize data analysis and visualization.

### Network Analysis

In this study, a gene ontology (GO) enrichment analysis (Gene Ontology, 2015) was performed to identify the function of our candidate genes. We investigated PDA-related genes using gene ontology (GO) terms enrichment scores of neighboring genes. By extracting important GO terms and pathways that can help us identify PDA-related genes. The enrichment theory of GO terms and pathways was adopted to encode each gene. Then, feature selection methods were employed to analyze these features and obtain the key GO terms and pathways. Then, we performed a protein-protein interaction (PPI) network analysis (De Las Rivas and Fontanillo, 2010), a bioinformatics analysis with the search tool for the retrieval of interacting genes/proteins program, to detect the key genes. Our candidate pathogenic genes with CNVs and the known disease-causing genes were uploaded in STRING database (Brohee et al., 2008), and PPI network was generated by Cytoscape software (Shannon et al., 2003). Known genes from previous literature and public databases, and they were divided into two different gene lists: (1) genes related to cardiac and vascular development; (2) genes related to PDA (Supplementary Table S1). We screened 20 genes from rare CNV and analyzed the network relationship between these genes and two gene lists.

### Expression Pattern in Human Embryonic Heart

The embryo can be classified according to its age, its size or its morphologic characteristics. The correlation between these three criteria will allow identifying the embryonic Carnegie stages. The 8 embryonic weeks (56 days) are divided into 23 Carnegie stages. The development of human embryo after implantation covers the most critical period of early embryo organ formation, that is, from Carnegie stage 10 to Carnegie stage 16 (Carlson, 2019). To detect the expression of our candidate genes in embryonic heart, we collected Human embryos from Carnegie stages 10–16 after medical termination of pregnancy at Shanghai Xin Hua Hospital. Human embryonic heart samples were remained for transcriptome array. The RNA was extracted by TissueLyserII (Qiagen) and the RNeasy MinElute Cleanup Kit (Qiagen). Then we used the Experion automated gel electrophoresis system (Bio-Rad, United States) and the NanoDrop 2000c spectrophotometer (Thermo Fisher Scientific, United States) to detect the integrity and purity of the RNA (Pfaffl, 2001). The Affymetrix HTA 2.0 microarray was used to detect the expression patterns of candidate genes in human embryonic heart.

## Results

### Clinical Characteristics

**FIGURE 1:**
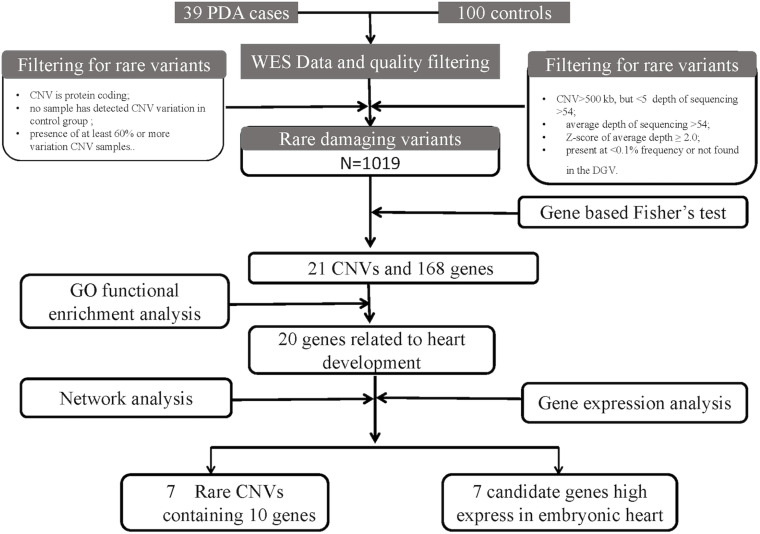
An analytical strategy workflow of the different steps taken during whole-exome sequencing (WES) analysis with gene expression detection is shown. Gene based fisher’s test, Gene ontology (GO) functional enrichment analysis, gene network analysis and microarray analysis were performed to identify our candidate genes.

The workflow is summarized in Figure 1. A cohort of 39 patients with PDA and 100 controls were enrolled. The average age of the patients in this study was 2.92 ± 2.44 years (range 0–13 years) and the gestational age is 39.04 ± 1.46 weeks. Among these patients, 15 patients are male, and 24 patients are female. And no one has multiple major developmental anomalies, major cytogenetic abnormalities, or developmental syndromes. CHD phenotypes included atrial septal defect (ASD, *n* = 7), ventricular septal defect (VSD, *n* = 2) (Table 1).

**TABLE 1:** Characteristics of 39 PDA Patients.

Patients characteristics	Numbers
Age	2.92 ± 2.44
Male n(%)	15 (38%)
Female n(%)	24 (61%)
Male-to-Female ration (%)	62%
BMI (kg/m2)	16.58 ± 4.34
PDA size (mm)	2.87 ± 1.68
Birth weight(kg)	2.96 ± 0.73
Gestational age(week)	39.04 ± 1.46
**Associated cardiac defect n (%)**	
VSD n(%)	2 (5%)
ASD n(%)	7 (18%)
Others n(%)	2 (5%)

*All values are expressed as mean ± SD or n (%).*

### Rare CNVs in PDA and Identification of Candidate Genes

**FIGURE 2:**
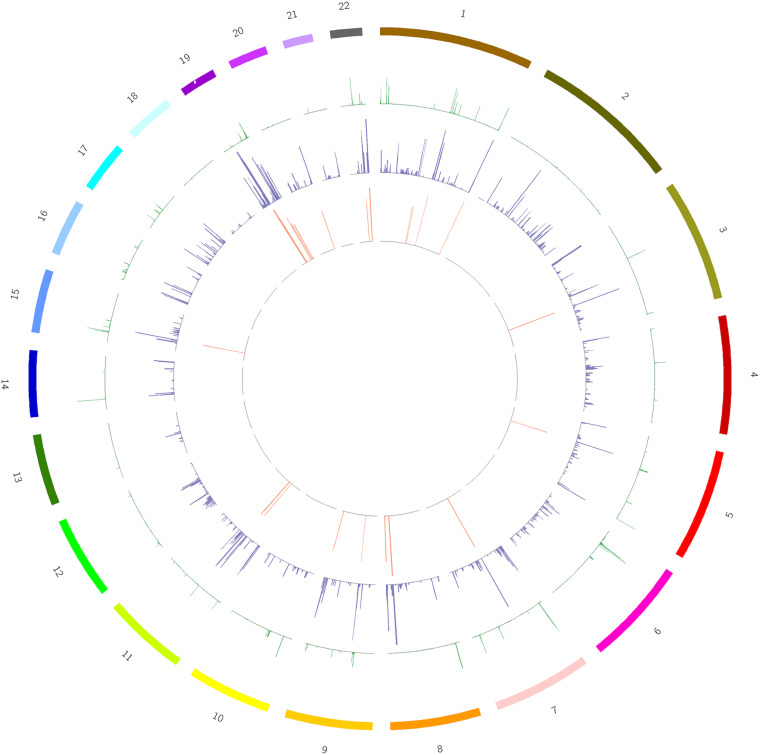
A circos plot depicts the chromosomal distribution, gene expression and copy number variations (CNV) in case and control groups. From outside to inside: Circle 1: Chromosomes, Circle 2: CNV in the control group, Circle 3: CNV in the case group, Circle 4: 168 genes screened out. The heights of circles 2, 3, and 4 represent the proportion of samples of CNV or genes in the corresponding group (CNV samples/total samples in the group).

**FIGURE 3:**
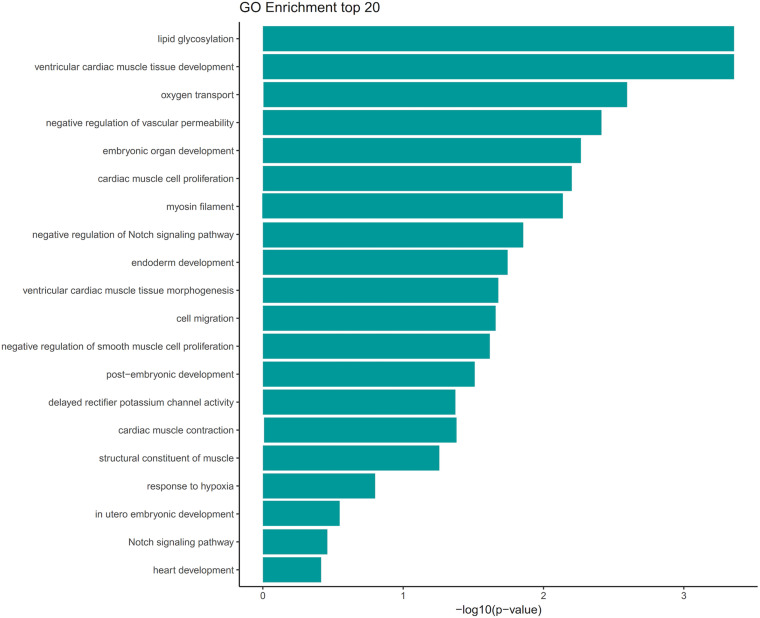
GO term analysis of the 168 genes showed enrichment of several categories associated with heart development and vascular development. GO terms were filtered for adjusted with *P* value < 0.05. Selected enriched GO terms were plotted.

We screened out rare CNVs by comparing with the CNV/SV dataset from 1000 Genomes Project (Supplementary Table S2). Using the strict CNV analysis strategy described in the Methods, 1019 cases of CNV were identified, of which 963 (95%) were duplications, and 56 (5%) were deletions (Supplementary Table S3). Then we used Fisher’s test to detect rare the pathogenic CNV candidates between the case and control group. 21 CNVs containing 168 candidate genes were filtered by Fisher’s test and 19 (90%) were duplications, and 2 (10%) were deletions. Then the CNV segments and genes classified as loss and gain were input to R package to generate a circos plot for visualization of recurrent CNV regions (Figure 2). The results showed that chromosome 1 and 19 had the most frequent gain-of-copy and loss-of-copy CNVs in our patients, respectively. Then we performed a GO functional enrichment analysis and identified the function of those genes (Figure 3). Among them, items related to heart development or embryonic development were significantly enriched, including heart development (GO:0007507), embryonic organ development (GO:0048568), negative regulation of vascular permeability (GO:0043116) and so on. And the main 20 genes involved is MYC, FOS, VAV2, WDR5 and so on (Table 2).

**TABLE 2:** 20 candidate genes by gene ontology (GO) functional enrichment analysis.

Gene	Chromosome	Start	End	GO-id	GO-term	Case-DUP	Case-DEL
PLXNA1	3	126707437	126756235	GO:0014910	regulation of smooth muscle cell migration	31	0
MAP3K1	5	56111401	56191979	GO:0030334	regulation of cell migration	0	24
ARC	8	143692405	143696833	GO:0007492	endoderm development	29	0
MYC	8	128747680	128753674	GO:0007219	Notch signaling pathway	37	1
TRIB1	8	126442563	126450647	GO:0048662	negative regulation of smooth muscle cell proliferation	38	0
RXRA	9	137208944	137332431	GO:0055010	ventricular cardiac muscle tissue morphogenesis	26	0
WDR5	9	137000487	137025093	GO:0051568	histone H3-K4 methylation	24	0
VAV2	9	136627016	136857726	GO:0016477	cell migration	24	0
OR5L2	11	55594695	55595630	GO:0004930	G-protein coupled receptor activity	24	0
SLC35C1	11	45825623	45834566	GO:0045746	negative regulation of Notch signaling pathway	26	0
TJP1	15	29991571	30261068	GO:0043116	negative regulation of vascular permeability	24	0
ERCC1	19	45910591	45982086	GO:0048568	embryonic organ development	25	0
ERCC2	19	45853095	45874176	GO:0009791	post-embryonic development	25	0
CD3EAP	19	45909467	45914024	GO:0009303	rRNA transcription	25	0
PPP1R13L	19	45882892	45909607	GO:0003229	ventricular cardiac muscle tissue development	25	0
MYBPC2	19	50936160	50969578	GO:0008307	structural constituent of muscle	24	0
FOSB	19	45971253	45978437	GO:0032870	cellular response to hormone stimulus	25	0
PSG3	19	43225790	43244721	GO:0007565	female pregnancy	31	0
KCNQ2	20	62037542	62103993	GO:0005251	delayed rectifier potassium channel activity	25	0
CHRNA4	20	61975420	62009753	GO:0001666	response to hypoxia	25	0

### Network Analysis

**FIGURE 4:**
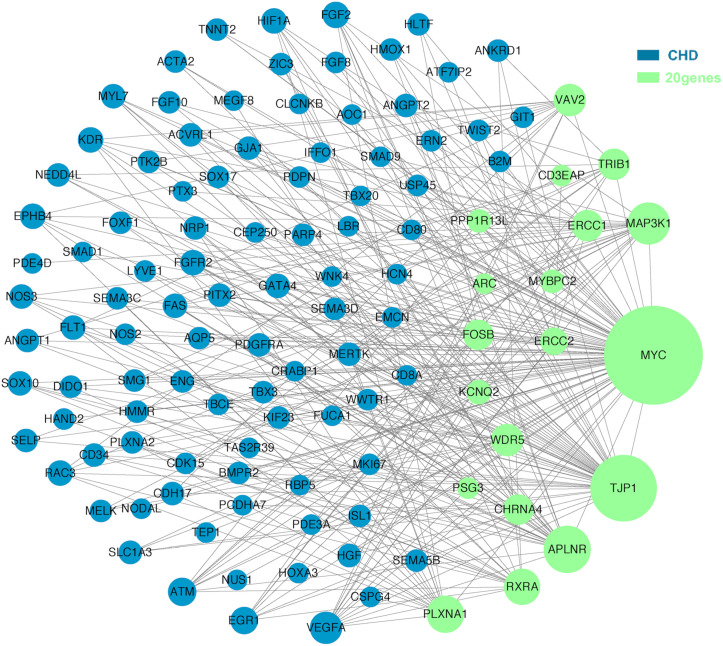
A protein-protein interaction (PPI) network was constructed between candidate genes and known pathogenetic genes of CHD. The green nodes represent rare CNVs loci genes in this study and the blue nodes represent the genes in list 1. The different size of the green nodes represent different intensity of the protein interaction, and the larger the green nodes, the closer the interaction is.

**FIGURE 5:**
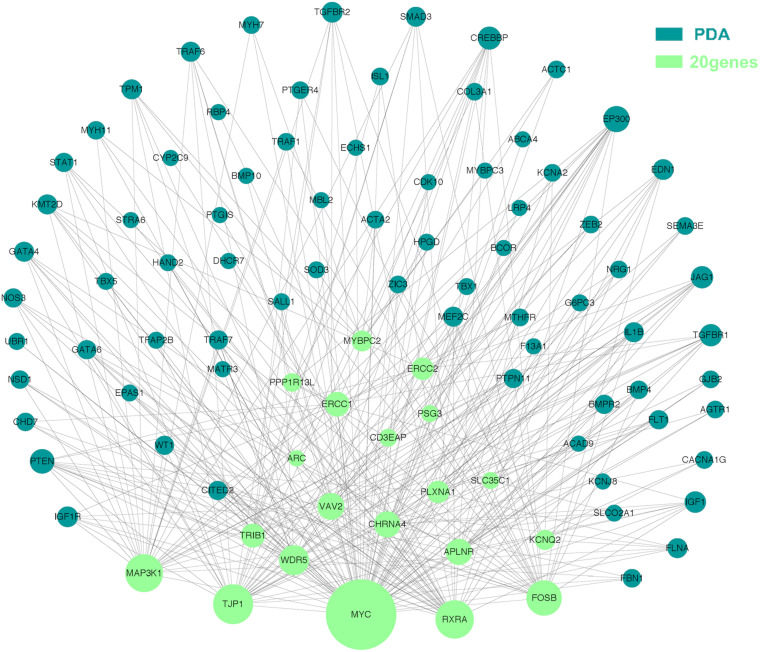
Utilizing integrated human PPI network between 20 candidate genes and known genes associated with PDA. The green nodes represent rare CNVs loci genes in this study and the blue nodes represent the genes in list 2. The different size of the green nodes represent different intensity of the protein interaction, and the larger the green nodes, the closer the interaction is.

The molecular mechanism of PDA formation is quite complicated. Various factors affect each other and participate in the occurrence of diseases together, including heart and vessel abnormally development, increase in vasoactive peptides and a decrease in prostaglandin E2 levels. In addition, multiple systemic syndromes also showed PDA, like Char syndrome, Loeys-Dietz syndrome and so on. Therefore, we got 244 known pathogenic genes from previous literature and publicly database. Then using STRING database to generate the PPI network between 20 candidate genes and two groups pathogenic genes, respectively (Figures 4, 5). And PPI network was generated by Cytoscape software. By analyzing the relationship between these candidate genes and two gene groups, we identified the finally 7 CNVs containing 10 pathogenic genes (MAP3K1, MYC, VAV2, WDR5, RXRA, APLNR, TJP1, ERCC2, FOSB, CHRNA4) (Table 3). And these CNVs were 5q11.2 deletion, 8q24.13 duplication, 9q34.2 duplication, 11q12.1 duplication, and 15q13.1 duplication, 19q13.32 duplication and 20q13.33 duplication. Among these rare CNVs, some of them are reported to be related to CHD. The 5q11.2 deletion previously detected in TOF children with growth and development disorders (Prescott et al., 2005). In addition, microduplications of 9q (9q +) have been described in patients with CHD and 9q34.2 duplication has been reported in relation to TOF in previous study (Amarillo et al., 2015). However, 8q24.13 duplication, 11q12.1 duplication, and 15q13.1 duplication, 19q13.32 duplication and 20q13.33 duplication observed in our study have never been reported to be related to congenital heart defect. Moreover, our data also suggested that these ten genes directly interacts with both two groups of genes and have strong roles in cardiac development and pathogenesis of PDA. And MAP3K1, MYC, TJP1 were the most obvious relation to known Congenital heart defect (CHD) pathogenic genes. Interestingly, these three genes also had strongly associated to known pathogenic genes of PDA.

**TABLE 3:** Copy number variant (CNV) in patients with PDA and identification of candidate genes.

Locus	Start	End	Size(bp)	CN	Genes
5q11.2	55271819	56160752	888933	Loss	MAP3K1
8q24.13-q24.21	126448254	128753230	2304976	Gain	MYC
9q34.2-q34.3	136399757	137777116	1377359	Gain	VAV2,WDR5,RXRA
11q11-q12.1	55321803	57081431	1759628	Gain	APLNR
15q13.1-q13.2	30260863	30845202	584339	Gain	TJP1
19q13.32	45766310	46333603	567293	Gain	ERCC2,FOSB
20q13.33	61588157	62197556	609399	Gain	CHRNA4

### Expression Pattern of Candidate Genes in Human Embryonic Heart

**FIGURE 6:**
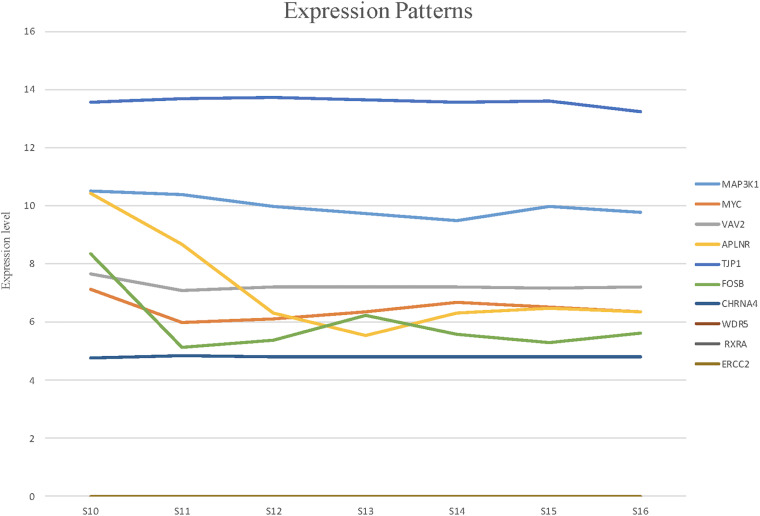
The expression patterns of candidate genes in human embryonic heart at different stages of S10 to S16 were analyzed by microarray. Among these candidate genes, the expression levels of CHRNA4 were significantly higher than those of other genes.

**FIGURE 7:**
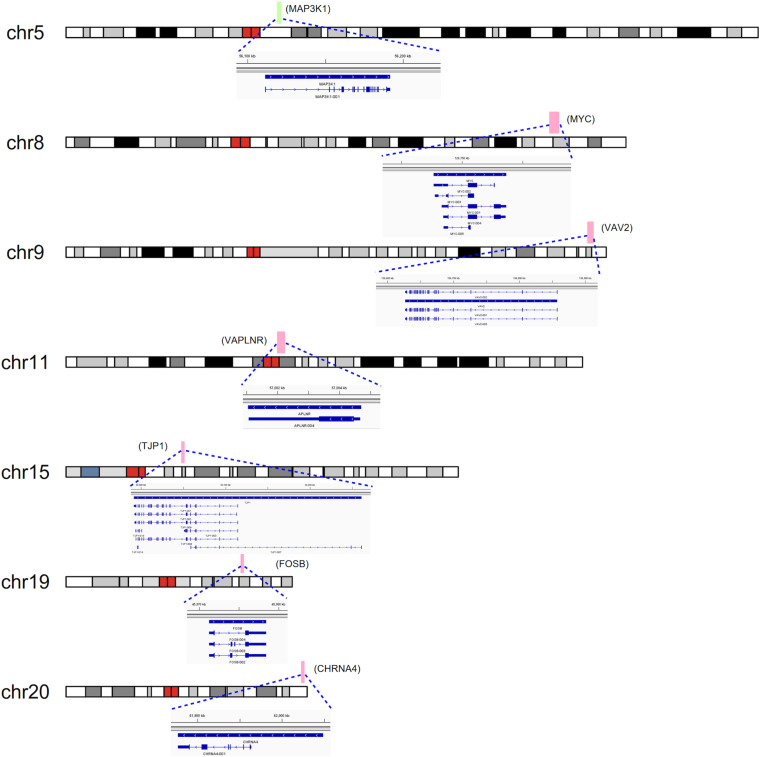
CNVs were analyzed with respect to their structural and genomic characteristics including span, and chromosomal location. Rare CNVs overlapping novel candidate gene for PDA: MAP3K1, MYC, VAV2, APLNR, TJP1, FOSB, and CHRNA4. The dotted rectangles represent the location of genes.

To detect the expression of these candidate genes in embryonic heart, we collected human embryonic heart in different Carnegie stages from S10 to S16. Then we performed the gene expression analysis using microarray (Figure 6). The results showed that the CHRNA4 expressed the most highly in embryonic heart. In addition, WDR5, RXRA, ERCC2 were not expressed in human embryonic heart. According to the results of embryo expression profile of the selected genes, we screened and identified the final seven pathogenic genes (MAP3K1, MYC, VAV2, APLNR, TJP1, FOSB, CHRNA4). Figure 7 represent the position of rare CNV identified in our patients with PDA containing the final seven pathogenic genes (Figure 7). These data suggested that those genes play important roles in cardiac development and pathogenesis of PDA.

## Discussion

Patent ductus arteriosus (PDA) is a common congenital heart defect (CHD), with a structure called ductus arteriosus (DA) connecting pulmonary artery and aorta after birth (Golombek and Sola, 2015). Persistent patency of the DA can lead to pulmonary hypertension, heart failure and even death (Chorne et al., 2007). Previous studies have shown that some genes are related to PDA (Hajj and Dagle, 2012). But the molecular mechanisms are still unclear. To detect the underlying pathogenetic mechanism of PDA, we recruited 39 patients with PDA and 100 healthy controls and performed Whole Exome Sequencing (WES) to identify the rare CNVs in PDA. In our study, we explored the clinical characteristics and genetic cause of PDA. We firstly identified 7 totally novel candidate genes (MAP3K1, MYC, VAV2, WDR5, RXRA, APLNR, TJP1, ERCC2, FOSB, CHRNA4) associated with PDA. In addition, using the STRING protein-protein interaction network analysis, we found that MAP3K1, MYC, TJP1 are strongly associated with known pathogenic genes. With the development of genetic testing technology for CHDs, deletion and duplication of CNV have become increasingly important in diagnosis and gene discovery. Although recent studies have demonstrated a role of newly occurring CNV mutations in PDA, the current understanding of the CNVs in the etiology of PDA is limited. Li et al. (2019) used CNV sequencing identified a 0.86 Mb duplication in the 22q11.2 region in just one patient with PDA. Other than that, there is no research about CNV in PDA. Therefore, in order to investigate the pathogenesis of PDA, we collected DNA samples from 39 patients with PDA and 100 controls. We identified rare or *de novo* genic deletions and duplications of CNV and seven of these CNVs appear to be pathogenic or potentially pathogenic to PDA.

In our study, a total of 25(25/39, 64%) patients in FOSB had a duplication which is located on chromosome 1q44. FOSB is one of the members of FOS family. FOSB has been considered as a regulator of cell proliferation, differentiation and transformation (Dobrazanski et al., 1991). The relationship between the genetic regulation of FOSB expression and cardiovascular development has not been reported and it might be a total new candidate gene for PDA.

A total of 24(24/39, 61%) patients in VAV2 had a duplication. VAV2, the second member of the VAV guanine nucleotide exchange factor family of oncogenes, is related to epidermal growth factor receptor binding and angiogenesis and located on chromosome 19p12. The VAV2-Rac1 pathway plays an important role in vasodilatation of vascular smooth muscle cells (VSMCs) (Fabbiano et al., 2014). Previous study has demonstrated that the migration of neural-crest-derived cells and transformation to vascular smooth muscle cells (VSMCs) are the anatomical mechanisms of PDA formation. And a study revealed that VAV2 is an important candidate gene for Total anomalous pulmonary venous connection (TAPVC) (Shi et al., 2018). Above all, we speculate that duplication of VAV2 may have contributed to our patient’s cardiac phenotype, however, additional studies are needed to determine how genetic perturbations of VAV2 contribute to PDA.

A total of 25 patients (25/39, 64%) have duplication in APLNR and 24 patients (24/39, 61%) had duplication in TJP1, both located on chromosome 1q21.1. Apelin receptor (APLNR) is an endogenous ligand of seven-transmembrane G-protein-coupled receptor. Apelin and APJ are distributed in various tissues, including the heart, lung, liver, kidney, and gastrointestinal tract and even in tumor tissues (Huang et al., 2019). APLNR has been widely reported to be involved in heart and vascular development and disease. A growing body of evidence now demonstrates a regulatory role for the apelin/APJ receptor system in cardiovascular physiology and pathophysiology (Huang et al., 2019). In our study, we found that the expression of APLNR is related to heart development (GO: 0007507) in GO enrichment analysis. Tight junction protein 1 (TJP1) encodes the multifunctional protein ZO-1, which comprises 4 different domains, PDZ, SH3, GUK, and ZU5. Previous study has shown that the tight junction protein ZO-1,encoded by TJP1, regulates cell migration, barrier formation of primary endothelial cells and angiogenesis (Tornavaca et al., 2015). And it has been reported that there is a causal relationship between TJP1 pathogenic variants and Arrhythmogenic Cardiomyopathy (De Bortoli et al., 2018). In our study, they might be newly associated with PDA pathogenesis and the relationship between APLNR, TJP1 and PDA needed to be further researched.

A total of 37 (37/39, 94%) patients had duplication and 1 (1/39, 2%) patients had deletion in MYC and a deletion in MAP3K1 was identified in 24 patients (24/39, 61%). MYC have the most patients than others. Both genes are located on chromosome 15q13.2. MYC proteins belong to the basic helix-loop-helix-domain family and exert their functions mainly by regulating transcription. Overexpression of MYC may enhances myocyte proliferation promoting cardiac hyperplasia during heart development in mice (Jackson et al., 1990). MYC is considered an essential transcription factor for heart development. MYC, a driver of anabolic metabolism and growth, was suppressed by forkhead box O(FOXO), leading to vessel thinning and hypobranching (Wilhelm et al., 2016). Mitogen-activated protein kinase kinase-1(MAP3K1) is a mitogen-activated protein kinase kinase kinase. Previous studies have suggested that the expression of MAP3K1 plays a key role in cardiac hypertrophy and apoptosis by MEKK1-JNK pathway. It is essential for cardiac hypertrophy and dysfunction (Sadoshima et al., 2002). Thus far, MYC and MAP3K1 may be new candidate genes in the pathogenesis of PDA, but the mechanism is not clear.

A total of 25 patients (25/39, 64%) have duplication in CHRNA4. The CHRNA4 encode the α4 subunits of the nicotinic acetylcholine receptors. In our study, the CHRNA4 expressed the most highly in embryonic heart. Several studies have indicated the CHRNA4 as strong candidates for the understanding of genetic factors related to epilepsy syndrome (Wang et al., 2018). And it is reported that CHRNA4 may participate in congestive heart failure (Andersson et al., 2006). The relationship between CHRNA4 and PDA needs to be further validated experimentally. It might be new gene for PDA pathogenesis and need more systematic investigations and studies.

In conclusion, we identified 7 CNVs and 7 candidate genes associated with PDA. Those genes are totally new and has not been reported in PDA. Based on our result, our findings open up new fields for PDA genetic research and provide new insights into understanding the pathogenesis of PDA. However, our study is limited. Lack of parental samples and small sample size are our limitations. We need larger or multicentric studies to further replication study. In addition, we need more fundamental research to identify the functions or potential mechanism of our candidate genes.

## Data Availability Statement

The datasets generated for this study can be found in NCBI SRA. The accession number is SRP288538(SRR12897411-SRR12897449).

## Ethics Statement

The studies involving human participants were reviewed and approved by the Medical Ethics Committee of Xinhua Hospital. Written informed consent to participate in this study was provided by the participants’ legal guardian/next of kin.

## Author Contributions

PZ and YG contributed to design of the study and performed the statistical analysis. AH, LZ, YL, XS, and BD collected the blood samples from all subjects. BC wrote the first draft of the manuscript. AH and BC contributed to this study equally. PZ and YG revised the manuscript. All authors contributed to manuscript revision, read and approved the submitted version.

## Conflict of Interest

The authors declare that the research was conducted in the absence of any commercial or financial relationships that could be construed as a potential conflict of interest.

## Footnotes

1 http://dgv.tcag.ca/
